# Australian dental students’ knowledge on antibiotics prophylaxis for dental procedures

**DOI:** 10.1186/s12903-022-02660-x

**Published:** 2022-12-23

**Authors:** Charn Thanissorn, Joon Soo Park, Kate N. Wang, Marc Tennant, Amy T. Page, Estie Kruger

**Affiliations:** 1 International Research Collaborative - Oral Health and Equity, The University of Western Australia, Crawley, WA Australia; 2 Institute for Sustainable Industries and Liveable Cities, Victoria University, Melbourne, VIC Australia; 3 School of Medicine, The University of Notre Dame Australia, Fremantle, WA Australia; 4 UWA Dental School, The University of Western Australia, Nedlands, WA Australia; 5 School of Health and Biomedical Sciences, RMIT University, Bundoora, VIC Australia; 6 School of Allied Health, University of Western Australia, Crawley, WA Australia

**Keywords:** Dental students, Competence, Pharmacotherapy, Prescribing, Antibiotic prophylaxis

## Abstract

**Background:**

Prescribing medicine is integral to clinical dentistry. Infective endocarditis may be rare but fatal if left untreated. As a result, judicious prescribing of antibiotics should be implemented due to potential. To our knowledge, no Australian study has examined dental students' knowledge and perceptions about antibiotic prophylaxis for dental procedures.

**Methods:**

Australian dental students were invited to undertake the survey comprising case vignettes to investigate their medication knowledge. A total of 117 responses were received. The questions were 12 clinically relevant questions and three perception-based questions. Results were analysed using descriptive statistics as well as the chi-squared test.

**Results:**

The 117 respondents had a mean correct response of 7.34 ± 2.64 (range 3–12 out of 12). Out of 117 students, 89 (76%) answered more than half of the questions correctly. Only three students (3%) answered all the questions correctly. Nearly two-thirds felt that they knew about antibiotic prophylaxis used for dental procedures.

**Conclusion:**

Most respondents answered more than half, but not all, of the clinical questions correctly. It is crucial to highlight that dental student may never receive any more training on antimicrobial stewardship (AMS) at any point in their future careers. It may be ideal that this issue is addressed at the dental school. One way to target this is to potentially nationalised teaching delivery of dental AMS across Australia.

**Supplementary Information:**

The online version contains supplementary material available at 10.1186/s12903-022-02660-x.

## Background

The global issue of antimicrobial resistance has risen dramatically, which has now been recognised by the World Health Organization (WHO) as a serious public health threat facing humanity [1]. Despite dentists prescribing up to 10% of antibiotics worldwide, oral health professionals have limited engagement in multisectoral antimicrobial resistance working groups and national action plans [2]. This has called for a multifaceted approach that includes antimicrobial stewardship (AMS)—the strategic management of appropriate antimicrobial use. Dental procedures account for a large proportion of antibiotic prescribing, and it is thus important that upcoming Australian dentists have predisposing knowledge on appropriate AMS for dentistry [3]. Dentists play an increasing role in antibiotic governance and AMS promotion in hospital settings and community practice.

A relatively uncommon condition, infective endocarditis is an infection within the heart affecting the inner lining and/or heart valves [4]. As it has been associated with bacteraemia induced due to dental procedures, antibiotics have historically been prescribed as a prophylactic measure [5]. Infective endocarditis represents an extremely low incidence rate in Australia, representing around 5 per 100,000 person-years, with similar global rates of 3–10 per 100,000 people [4, 6]. However, despite its rarity, it brings a substantially high mortality rate [6–8]. Since mortality is so high, the key is to prevent it if it does occur.

The management of infective endocarditis is challenging. The bacteraemia from regular oral hygiene procedures such as toothbrushing is greater than that of an invasive dental procedure [9]. This emphasises the importance of maintaining good oral hygiene to reduce the incidence of bacteraemia from daily oral activities. However, its relative infrequency renders high-quality studies impractical, and infective endocarditis remains a disease that is non-specific and highly variable in disease presentation and course [4, 10]. Infective endocarditis also continues to evolve. Recent times have shown a shift in the predominant pathogens present [11, 12].

Guidelines for antibiotic prophylaxis, which have changed over the years, have thus primarily been based on expert consensus. There has been a progressive reduction in antibiotics recommended for infective endocarditis globally. There was the complete abolition of routine antibiotic prophylaxis in the United Kingdom following the recommendations of The National Institute for Health and Care Excellence in 2008 [13, 14]. More recently, there has been an updated 2020 evidence-based Good Practice Guidelines from the Faculty of General Dental Practice (UK) and the Faculty of Dental Surgery [15]. Australia and other committees around the world, such as the USA and Europe, have continued to recommend prophylactic cover for selected patients [16–19]. Despite its efforts to limit the number of conditions requiring antibiotic coverage, in 2015–2021, Australian dentists were responsible for almost 7 million antibiotic prescriptions dispensed [3]. However, according to a survey conducted, approximately 80% of overprescribing antibiotics was detected by general dentists [20]. Nevertheless, the authors further stated that recent graduates (0–5 years) generally scored better than their colleagues for antibiotic prescribing (*p* < 0.05).

In Australia, there were 7 million dispensed antibiotics, which equated to an average of 24 prescriptions per year per dentist [3]. Nevertheless, according to a population-level analysis of antibiotic prescription in 2017, dentists in Australia had the lowest antibiotic prescribing rate per 100 population compared to the United States, England, and British Columbia (Canada) [21]. However, the importance of AMS has to be brought to attention. Therefore, knowing the correct dosage, duration, and clinical scenarios to prevent infective endocarditis is essential. In Australia, these are presented in the Therapeutic Guidelines [16, 17]. However, to our knowledge, no Australian study has looked at dental students' knowledge and perceptions about antibiotic prophylaxis for dental procedures. Therefore, this study aims to investigate dental students' knowledge and attitudes towards antibiotic prophylaxis for infective endocarditis.

## Methodology

This study methodology was adopted from previously published studies [22, 27]. It is reported according to the Checklist for Reporting Results of Internet E-Surveys (CHERRIES) [23].

### Ethics

Ethics approval to conduct this case vignette study was obtained from the Human Research Ethics Committee at the University of Western Australia (Approval Number-2021/ET000120).

### Population

In Australia, nine dental schools offer dental programs accredited by the Australian Dental Council [24]. Three of the nine dental schools offer a four-year graduate entry programme, whereas the remaining six offer a five-year direct entry undergraduate programme [24]. Students undertake clinically supervised practice in the latter half of their programme after being deemed pre-clinically competent in simulated scenarios. In 2018, approximately 650 dental graduates across all Australian dental Schools [25, 26].

### Survey design

An anonymous survey was distributed to all dental students in the final two years of their degree. Survey questionnaires were available via a link and consisted of demographic details [gender, age, dental school attending, primary dental qualification, year level], twelve case vignettes, and three sliding scale opinion-based questions (0 to 100). Opinion-based questions assessed students' attitudes and perceptions of antibiotic prophylaxis. The full text of the questionnaire has been provided in Additional file 1.

### Case vignettes

Questions were formatted around clinical scenarios that would be assumed knowledge for graduate dental clinicians. Vignette-style questions on the appropriate use of antibiotic prophylaxis for preventing infective endocarditis in a wide range of clinical scenarios were included in the questionnaire. These questions were created using resources following the Australian Therapeutic Guidelines [Antibiotics] [17]. Two registered Australian pharmacists and registered Australian dentists verified the questionnaire content.

### Survey administration

The surveys were delivered through Qualtrics®^XM^ software (Provo, UT, USA) using an anonymous online link and were available over four months (10/02/2021–21/06/2021). A key staff member was contacted at each Australian dental school and provided information about the study and its aim. After obtaining permission, each staff member disseminated the online link through their email portals to their respective dental students. In addition, dental student representatives from each dental school were contacted to send the link via social media groups. The link and study information was also provided through relevant social media (Facebook, LinkedIn and Twitter). All completed anonymous questionnaires were returned (online) directly to the researchers. Monthly reminder follow-up emails and social media prompts were sent to ensure a timely response. For quality assurance, in addition to the 'Prevent Ballot Box Stuffing' in Qualtrics®^XM^ software, IP addresses were manually checked to identify potential duplicate entries from the same user.

### Statistical analysis

Normally distributed demographics and the number of students correctly answered the various questions were presented in both counts and percentages. Except for the sliding scale answers, all the responses to the questions were categorical as set out by the multiple-choice question nature of the case vignettes. The correct and incorrect responses were dichotomised. To compare outcomes of the dichotomised variables across gender and year levels, Pearson Chi-square tests were used. SPSS® version 27.0 (IBM Company, Chicago, IL, USA) was used, and the statistical significance was set at P < 0.05.

## Results

### Demographics

The questionnaire was completed by 117 invited students (Table 1). However, 47 questionnaires could not be included as it was partially completed. The average time students completed the questionnaire was 7 minutes. Most responses (n = 75, 64%) were from female students.

**Table 1:** Demographics of Australian Dental students that participated in the questionnaire (N = 117)

Demographics	Count (%)
Gender	
Male	41 (35)
Female	75 (64)
Other	1 (1)
Year level	
UG (Year 4—Penultimate)	28 (24)
UG (Year 5—Final)	18 (15)
PG (Year 3—Penultimate)	27 (23)
PG (Year 4—Final)	44 (38)
Dental school	
Charles Sturt University	3 (3)
Griffith University	5 (4)
James Cook University	7 (6)
La Trobe University	8 (7)
The University of Western Australia	28 (24)
University of Adelaide	9 (8)
University of Melbourne	44 (38)
University of Queensland	7 (6)
University of Sydney	6 (5)
Primary dentistry qualification	
Undergraduate dental degree (UG) (e.g. BDS, BDSc)	40 (34)
Postgraduate dental degree (PG) (e.g. DMD, DDS)	77 (66)

*UG* undergraduate, *PG* postgraduate

### Clinical knowledge of antibiotic prophylaxis prescribing

The mean number of correct responses was 7.34 ± 2.64 (range 3–12). Out of 117 students, 89 (76%) answered more than half of the questions correctly. Only three students (3%) answered all the questions correctly. The case vignettes where less than half of the dental students answered correctly were transcatheter-implanted prosthesis, nil indication of prophylaxis [scenario 1], and nil indication of prophylaxis [scenario 2] (Table 2). No statistical differences among dental students’ demographic variables (gender, year level, primary dental qualification) were noted in response to the questions on cephalexin dose, transcatheter implanted prosthesis, rheumatic heart disease, and nil indication of prophylaxis [scenario 2] (Table 2).

**Table 2:** Clinical knowledge of antibiotic prophylaxis prescribing for dental students (N = 117)

Topic	Count (%)	Pearson Chi-Square
Dosage and timing	103 (88%)	Female students answered better (*p* < 0.001)
Route of administration	93 (79%)	
Dose—penicillin allergy alternative—cephalexin	63 (54%)	
Dose—penicillin allergy alternative—clindamycin	88 (75%)	Male students answered better (*p* < 0.001) Undergraduate degree students answered better (*p* = 0.005) Undergraduate penultimate year students answered better (*p* = 0.025)
Valvular replacement	99 (85%)	Female students answered better (*p* = 0.046)
Transcatheter-implanted prosthesis	32 (27%)	
Previous history of infective endocarditis	107 (91%)	Female students answered better (*p* < 0.001)
Congenital heart disease	94 (80%)	Female students answered better (*p* = 0.002)
Rheumatic heart disease in high-risk patients	94 (80%)	
Nil indication of prophylaxis—scenario 1	33 (28%)	Female students answered better (*p* = 0.049) Postgraduate final year students answered better (*p* = 0.021)
Nil indication of prophylaxis 2—scenario 2	44 (38%)	
Nil indication of prophylaxis 3—scenario 2	70 (60%)	Postgraduate penultimate year students answered better (*p* = 0.023)

### Self-reported perception of antibiotic prophylaxis prescribing knowledge

Dental students' self-reported knowledge of antibiotic prophylaxis prescribing was generally positive (Table 3). Nearly two-thirds [mean (± standard deviation): 64 (SD 22)] felt that they had knowledge regarding antibiotic prophylaxis used for dental procedures. Furthermore, nearly two-thirds [mean (± standard deviation): 60 (SD 22)] were confident that they would be able to prescribe safe and effective antibiotic prophylaxis for their patients. In addition, nearly three out of four dental students [mean (± standard deviation): 74 (SD 27)] stated a need for further education in appropriate antibiotic prescribing in the dental curriculum.

**Table 3:** Perception of antibiotic prophylaxis prescribing knowledge for dental students (N = 117)

Perspectives	Mean% (± standard deviation)
Knowledge	64 (± 22)
Confidence	60 (± 26)
Necessity	72 (± 27)

## Discussion

This study aimed to further elucidate the knowledge and perceptions of antibiotic prophylaxis for dental students in their clinical years within an Australian context. To our knowledge, this is the first to explore this in Australia. Overall, the students correctly answered less than two-thirds of the total questions. This was similar to the results obtained in the previous case-vignette study [27]. Despite students' generally positive self-reported perception of antibiotic prophylaxis knowledge and suitable prescribing capability, many felt a need to further reinforce this knowledge in dental curriculums. In an international context, according to the survey assessing the endocarditis prophylaxis knowledge of Flemish dentist and paediatricians, it was evident that younger dentist was better aware of recent guidelines underlining improved teaching [28]. Nevertheless, there was an increase in antibiotic prescribing amongst the younger Flemish dentists not because of a lack of knowledge about dental procedures but because of a lack of knowledge of low and high-risk types of congenital heart disease.

It was interesting to note that students performed on topics of dosage and timing, as well as scenarios indicating prophylaxis. This is reflected by 80% of the students knowing when to correctly prescribe in four of the five appropriate scenarios. This was also indicative of the last study conducted [27]. On the contrary, scenarios that were seen as inappropriate for prophylaxis had a markedly lower performance, which could be reflective of the overuse of antibiotics among general dental practitioners. It is important to highlight that there could be potentially detrimental outcomes for the patients with a recently implanted device (i.e., pulmonic valve, ventricular septal defect device), but it also leads to overuse of antibiotics, which is a problem in the struggle against antibiotic resistance. The recognition of previous infective endocarditis was also particularly high, highlighted in comparable international studies looking at appropriate antibiotic prophylaxis prescriptions amongst dental students [29, 30]. Outside of this, it was difficult to compare such studies considering that they had used the AHA guidelines as their guiding principle in management, which differs from the Australian approach [31]. In 2012, Swedish Medical Products Agency removed the prophylactic administration of antibiotics at risk of contracting infective endocarditis caused by oral viridans group streptococci (VGS) during certain dental procedures [32]. Regardless, a recently published longitudinal study showed no increase in infective endocarditis despite the cessation [32]. This further highlights the ongoing debate that dental students must be aware of when it comes to pharmacotherapeutic education.

The perception of a need for further education on antimicrobial use in dental school raises the question of whether enough is currently being implemented within curricula in Australia to educate future dentists. Having been described to have applications within numerous specialities of dentistry, one would reasonably assume that antibiotics are a well-traversed topic. It could potentially be suggested that the reason for this is that there is no cohesive training on antimicrobial stewardship in a broad sense, such as a comprehensive AMS module which has shown to be receptive by students internationally [33]. This could be something that could be considered in Australian dental schools.

Graduation from dental school in Australia allows dentists to work autonomously. Dentists are given the privilege of being able to prescribe various medicines without having to undergo an internship or training year to receive a general registration [34]. This largely contrasts with other primary and allied health fields where further training is mandatory. This means that the last time Australian dentists could be didactically taught the principles of antimicrobial stewardship without seeking the information themselves was in dental school. This once again highlights the need for rigorous nationalised AMS training across Australian Dental Schools.

This study has several strengths and limitations. Firstly, the case vignettes utilised Therapeutic Guidelines set by the Australian standards, which were set by multi-disciplinary teams of health professionals. For one, the questionnaire was available to students over four months, with a multichannel approach to improve questionnaire response rates through social media, emails and notifications from their relevant dental student and education bodies. The lack of restrictions upon timing and access to external information and resources emulated a real-world approach in which clinicians are not solely reliant on past memorisation and can access other resources. Despite this, the study still had a relatively low sample size, heavily represented by two of the nine dental schools. This could lead to sampling bias. This made comparisons between various dental schools not possible, nor was it the purpose of this study as we aimed to look at Australian students.

The result of this study provides valuable information on current dental students and their perception of antibiotic prophylaxis for dental procedures. In addition, it highlights the bigger picture to recognise that dentists will never be able to discriminate fully between low and high-risk cardiovascular disease because of the lack of in-depth knowledge of the field [35, 36]. Therefore, there could be a potential avenue for optimal patient care if there is good interaction between dentists, patients, and cardiologists/paediatricians/general physicians to provide a tailored individualised treatment plan. Future studies could elucidate a relationship, contrast students' knowledge with registered dentists, or compare and contrast the understanding between different schools. In addition, this study could create a dialogue for potential nationalised teaching delivery across Australian Dental Schools.

## Conclusion

Australian dental students who participated showed a greater ability to correctly identify scenarios indicated for antibiotic prophylaxis than contraindicated scenarios. However, some respondents in this study could not correctly answer questions about antibiotic prophylaxis prescribing in various clinical scenarios. Their perceptions of antibiotic prophylaxis were generally positive, but many acknowledge a need for further education. Future research should be undertaken to determine whether professional development in pharmacotherapeutics or curriculum redesign is warranted.

## Supplementary Information

**Additional file 1.** Questions of antibiotic prophylaxis pharmacotherapeutics for dental students.

## Data Availability

The datasets generated and/or analysed during the current study are not publicly available due relevancy of the study but are available from the corresponding author on reasonable request.

## Abbreviation

AMS: Antimicrobial stewardship
