# Supplementary material for: Australian dental students’ knowledge on antibiotics prophylaxis for dental procedures
Source: BMC Oral Health. 2022 Dec 23;22:633. doi: 10.1186/s12903-022-02660-x (PMC9783433; doi:10.1186/s12903-022-02660-x)
Supplement: Supplementary file 1 — Additional file 1. Questions of antibiotic prophylaxis pharmacotherapeutics for dental students. [file 12903_2022_2660_MOESM1_ESM.docx]

Additional file 1 - Questions of antibiotic prophylaxis pharmacotherapeutics for dental students

Case Vignettes - Dose/Dosage

These sets of questions will assess your knowledge of antibiotic prophylaxis dosage

What is the ideal dose/dosage (and timing) of Amoxicillin for a case requiring antibiotic prophylaxis [Adult with no known medical condition]?

- 2g orally, 120 minutes before the procedure
- 2g orally, 60 minutes before the procedure
- 4g orally, 60 minutes before the procedure
- 4g orally, 120 minutes before the procedure
- Don't know

If oral administration of prophylaxis is not possible, what route of administration should be employed [Adult with no known medical condition]?

- Ocular route
- Intravenous and/or intramuscular route
- Transdermal route
- Buccal route
- Otic route
- Don't know

For patients with delayed nonsevere hypersensitivity to penicillins, what dose/dosage (and timing) of cefalexin should be provided for a case requiring antibiotic prophylaxis [Adult with no other known medical condition]?

- 2g orally, 120 minutes before the procedure
- 3g orally, 60 minutes before the procedure
- 2g orally, 60 minutes before the procedure
- 3g orally, 120 minutes before the procedure
- Don't know

For patients with immediate (severe or nonsevere) or delayed severe hypersensitivity to penicillins, what dose/dosage (and timing) of clindamycin should be provided for a case requiring antibiotic prophylaxis [Adult with no other known medical condition]?

- 600mg orally, 60 to 120 minutes before the procedure
- 300mg orally, 60 to 120 minutes before the procedure
- 600mg orally, 180 minutes before the procedure
- 300mg orally, 180 minutes before the procedure
- Don't know

Case Vignettes - Scenarios

These questions will assess whether antibiotic prophylaxis is required to prevent infective endocarditis (Please choose the most correct answer):

Which of the procedures require antibiotic prophylaxis?

- Patient with a prosthetic cardiac valve, undergoing a dental extraction
- Patient with a prosthetic cardiac valve, undergoing a comprehensive dental examination
- Patient with annuloplasty rings and chords, undergoing a simple filling
- None of the above
- Don't know

Which of the procedures require antibiotic prophylaxis?

- Patient with controlled diabetes, undergoing a biopsy
- Patient with a history of previous infective endocarditis, undergoing a deep scaling and clean requiring local anaesthetics
- Patient with hypertension, undergoing replantation of an avulsed tooth
- None of the above
- Don't know

Which of the procedures require antibiotic prophylaxis?

- Patient with rheumatic heart disease living in a low socioeconomic area, undergoing a composite restoration
- Patient with hypertension, undergoing a full clearance
- Patient living in a low socioeconomic status setting with rheumatic heart disease, undergoing a biopsy for a tongue
- None of the above
- Don't know

Which of the procedures DO NOT require antibiotic prophylaxis?

- Patient with a repaired defect adjacent to the site of a prosthetic device (which inhibits endothelialisation), undergoing wisdom tooth extraction
- Patient with a history of previous infective endocarditis, undergoing a deep scaling and clean requiring local anaesthetics
- Patient with a transcatheter-implanted prosthesis, undergoing implant placement
- None of the above
- Don't know

Which of the procedures require antibiotic prophylaxis?

- Patient with a pacemaker, undergoing an alveoplasty
- Patient with an unrepaired cyanotic defect, undergoing a tooth extraction
- Patient with annuloplasty rings and cords, undergoing fissure sealants
- None of the above
- Don't know

Which of the procedures require antibiotic prophylaxis?

- Patient with a transcatheter-implanted prosthesis, undergoing implant placement
- Patient with aortic valve surgery, who is having an avulsed tooth (knocked out tooth) replanted
- Patient with rheumatic heart disease living in a high socioeconomic area, undergoing facial reconstruction due to trauma
- None of the above
- Don't know

Which of the procedures DO NOT require antibiotic prophylaxis?

- Patient with a prosthetic cardiac valve, undergoing periodontal (gum) surgery
- Patient with a history of previous infective endocarditis, undergoing root canal
- Patient with annuloplasty rings and cords, undergoing a periapical (surgical root canal) procedure
- None of the above
- Don't know

Which of the procedures require antibiotic prophylaxis?

- Patient with a pacemaker, undergoing implant placement
- Patient with a cardiac stent, undergoing a biopsy
- Patient with no known medical conditions, undergoing multiple dental extractions
- None of the above
- Don't know

Knowledge, confidence, and attitude

One the scale 0 (no) to 100 (yes), please rate.

Do you have ANY knowledge regarding antibiotic prophylaxis used for dental procedures?

|  | 0 | 10 | 20 | 30 | 40 | 50 | 60 | 70 | 80 | 90 | 100 |
| --- | --- | --- | --- | --- | --- | --- | --- | --- | --- | --- | --- |

| Knowledge | 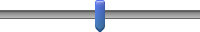 |
| --- | --- |

When it comes to ANTIBIOTIC PROPHYLAXIS FOR DENTAL PROCEDURES, I am CONFIDENT that I will be able to prescribe SAFE AND EFFECTIVE medications for my patients.

|  | 0 | 10 | 20 | 30 | 40 | 50 | 60 | 70 | 80 | 90 | 100 |
| --- | --- | --- | --- | --- | --- | --- | --- | --- | --- | --- | --- |

| Confidence | 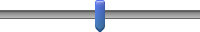 |
| --- | --- |

Further education in appropriate antibiotic prescribing in dentistry is NEEDED for the dental curriculum.

|  | 0 | 10 | 20 | 30 | 40 | 50 | 60 | 70 | 80 | 90 | 100 |
| --- | --- | --- | --- | --- | --- | --- | --- | --- | --- | --- | --- |

| Education | 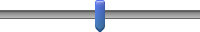 |
| --- | --- |
